# Supplementary material for: Hepatitis B and HIV coinfection in Northern Uganda: Is a decline in HBV prevalence on the horizon?
Source: PLoS One. 2020 Nov 18;15(11):e0242278. doi: 10.1371/journal.pone.0242278 (PMC7673526; doi:10.1371/journal.pone.0242278)
Supplement: S1 File — (PDF) [file pone.0242278.s002.pdf]

LACOR
Institutional Research Ethics Committee
APPROVED
VALID UNTIL
Date: [illegible]
P O Box 180 - Gulu

Prevalence and risk factors for HBV infection in HIV patients of Lacor Hospital, Uganda [PATIENT]

Date __/__/____

Patient's ID: ………………………………………………… ART number: ……………………………………………………
Name: ………
Date of birth: ……………………………………………… Age: …………………………………………………………
Village: …………………………………………………… Parish: ………………………………………………………
Sub county: …………………………………………………

1.Have you lived in an internal refugee camp? ☐Yes☐No If so, wich one? ……………………………………

2.Where do you lived in your first ten years of live? Village ……………………………………………
Parish ……………………………………………………
Sub county ……………………………………………

3.Gender: ☐male ☐female

4.Tribe: ☐Acholi ☐Lango ☐Madi ☐Baganda ☐Jalwo ☐Karimojong ☐Banyoro ☐Other

5.Religion: ☐Catholic ☐Pentecostal ☐Anglican/Protestant ☐Muslim ☐Other (…………)

6.Education: ☐no formal education ☐completed primary school ☐completed senior four ☐completed senior six ☐qualification after senior six

7.Employment: ☐ peasant farmer ☐informal employment (………………………………)
☐formal employment (……………………………) ☐not working

8.Have you ever been a soldier: ☐Yes ☐No

9.Marital status:☐single ☐married/cohabiting ☐divorced/separated ☐widow/widowed

10.How many spouses do you have? ☐monogamous ☐polygamous

11.Number of children: ……………… 12.How many brothers/sisters do you have? ……………………

13.How many people shared the house with you in your first 10 years of life? ………………………

14.How was the habitation you lived in during your first 10 years of life? Roof: ☐grass ☐iron
Floor:☐Earth/sand ☐cement/brick

15.How where you delivered? ☐Natural childbirth ☐Caesarean section ☐Don't know

16.Does your mother have Hepatitis B? ☐Yes ☐No ☐Don't know

17.Was there anyone known to have Hepatitis B in the compound where you grow up?☐Yes ☐No☐Don't know

18.Have you ever undergone therapeutic scarification practice (medicine applied throw skin cutting, "ebino", tea-tea)
☐Yes ☐No

19.Number of lifetime sexual partners: ……………………… 20.Are you circumcised? ☐Yes ☐No

21.Do you use injection drugs? ☐Yes ☐No

22.Previous blood transfusions? ☐Yes ☐No

23.Have you ever been a sex worker? ☐Yes ☐No

LACOR
Institutional Research Ethics Committee
APPROVED
VALID UNTIL
Date: 11-6-2016
P.O Box 180 - Gulu

[INTERVIEWER]

Patient's ID/ART number:

1. **HBV information**

HBsAg: ☐Positive ☐Negative — date of HBsAg test: __/__/____
AST: U/l
ALT: U/l — date of AST/ALT test: __/__/____

2. **HIV information**

Date of HIV diagnosis: __/__/____

CD4 cell at diagnosis:	cells/µl	date: __/__/____
CD4 cell at beginning of therapy:	cells/µl	date: __/__/____
CD4 cell at first check (6month):	cells/µl	date: __/__/____
CD4 cell at second check (12 month):	cells/µl	date: __/__/____
CD4 cell at third check:	cells/l	date: __/__/____
More recent CD4 cell:	cells/l	date: __/__/____
HIV viral load:	10^6/l	date: __/__/____

3. **HIV therapy**

In therapy for HIV? ☐Yes ☐No

First therapeutic regimen:	start date __/__/____	drug used:
Second therapeutic regimen:	start date __/__/____	drug used:
Third therapeutic regimen:	start date __/__/____	drug used:

Has the patient skipped some pills? ☐No ☐Yes, in the last week
☐Yes, in the last month ☐Yes, in the last year
☐Yes, he has skipped appointment in the past

4. **AIDS information**

AIDS: ☐Yes ☐No

WHO stage at enrolment:	☐1	☐2	☐3	☐4	Date: __/__/____
WHO stage at ART start:	☐1	☐2	☐3	☐4	Date: __/__/____
WHO stage actual:	☐1	☐2	☐3	☐4	Date: __/__/____

Note:
