# Supplementary material for: Hepatitis B and HIV coinfection in Northern Uganda: Is a decline in HBV prevalence on the horizon?
Source: PLoS One. 2020 Nov 18;15(11):e0242278. doi: 10.1371/journal.pone.0242278 (PMC7673526; doi:10.1371/journal.pone.0242278)
Supplement: S2 File — (PDF) [file pone.0242278.s003.pdf]

LACOR
Institutional Research Ethics Committee
APPROVED
VALID UNTIL
Date:........11-4-2017........
P.O Box 180 - Gulu

Prevalence and risk factors for HBV infection in HIV patients of Lacor Hospital, Uganda
[PATIENT]

Date __/__/____

Patient's ID: .... ART number: ....
Name: ....
Mwakani tye adi? .... Kinywali inino dwe adi? ....
Gangwu tye kwene? .... Parish ma megwu kono tye kwene? ....
Sub county kono? ....

1. In ibedo I kem ikare me lweny? ☐Eyo ☐Pe Kem mene? ....

2. I bedo kwene i acakki me kwoni pi kare me mwaka 10? Gangwu tye kwen ....
Parish ....
Sub county ....

3.Laco/Dako: ☐male ☐female

4.Kakani kono: ☐Acholi ☐Lango ☐Madi ☐Baganda ☐Jalwo ☐Karimojong ☐Banyoro ☐Other

5.Dini ni kono? ☐Katoli ☐Morokole ☐Ceemec/Purutanti ☐Cilam ☐Mukene (................)

6. Kwanni ogik kakwene? ☐pe akwano ☐Ityeko Kwan Praimari ☐Ityeko Klass angwen ☐Ityeko Klass abicel ☐Ityeko cinia abicel

7. Ityo tic ango? ☐ Lapur madit calo farm ☐ Pe latic mucara (....................................) ☐latic mucara (................................) ☐pe tye

8. Kong ibedo lamony: ☐Eyo ☐Pe

9. Itye ki dako/laco: ☐atye kena ☐ Anyomme-atye ki laco/dako ☐kinwa opoke ☐datoo/coo too

10. Ibedo ki mon/coo adi ikwoni? ....

11. Itye ki litino adi? .... 12.Itye ki omegini ki lomegini adi? ....

13. I bedo ki dano adi i gangwu ikare me mwaka apar me kwoni?..........................

14. I kare me mwaka apa me kwoni,ibedo i kit ot mene? Wi Ot: ☐Lum ☐Bati Dye Ot:☐Lobo/Opuyu ☐cementi/Matafali

15. Kinywali nining? ☐ Labongo ayela ☐ Kibara(Caesarean) ☐ Pe Angeyo

16. Mamani tye ki two Hepatitis B? ☐Eyo ☐Pe ☐ Pe Angeyo

17. I bedo kin ngat mo ma tye ki two Hepatitis B igang kam idongo iye? ☐Eyo ☐Pe☐Pe angeyo

18. Kare ma in latin,gwoknyo ki baro in calo pi lok me "ebino",tea tea, nyig kal? ☐Eyo ☐Pe

19. Wel dano /lorem i mar onyo lok me mit: ....

20. Kiliro komi me coo? ☐Eyo ☐Pe

21. I tiyo ki libira pi nongo yat/Itucce kekeni?☐Eyo ☐Pe

22. Kimedo remo ikomi? ☐Eyo ☐Pe

23. I temo tic me cato komi?☐Eyo ☐Pe
