# Supplementary material for: Hepatitis B and HIV coinfection in Northern Uganda: Is a decline in HBV prevalence on the horizon?
Source: PLoS One. 2020 Nov 18;15(11):e0242278. doi: 10.1371/journal.pone.0242278 (PMC7673526; doi:10.1371/journal.pone.0242278)
Supplement: Attachment — Submitted filename: Response to reviewers.docx [file pone.0242278.s004.docx]

St. Mary’s Hospital Lacor,

P. o. Box 180, Gulu

20^th^ September 2020

The Academic Editor,

Plos One

Dear Dr. Jason Blackard,

RE: RESPONSE TO REVIEW COMMENTS ON HEPATITIS B AND HIV COINFECTION STUDY IN UGANDA (**PONE-D-20-20265)**

We sincerely thank the Editor and the Reviewers for the very constructive comments given to us for rebuttal. Here below, in Tabular format, we have responded to all the concerns, point by point, showing the location of effected changes in the tracked and the clean manuscripts.

Once again, we look forward to your feedback.

Thank you once again,

Sincerely yours,

Dr. Emmanuel Ochola (On behalf of the Authors)

**Table of response to Reviewer Comments**

| **ISSUE** | RESPONSE | **Page/Line in tracked document** | **Final document** |
| --- | --- | --- | --- |
| **Reviewer: Editor** |  |  |  |
| 1. Please ensure that your manuscript meets PLOS ONE's style requirements, including those for file naming | The file and supporting information naming as well as style have been modified to fit Plos One requirements. | S1-S3  Whole document | S1-S3, whole document |
| Thank you for stating in the text of your manuscript "Written informed consent was obtained from all the participants or, in the case of those aged 13-17 years, their legal guardians". Please also add this information to your ethics statement in the online submission form | This information has been added to the ethics statement in the online submission form. | Ethics statement, submission portal | Ethics statement, submission portal |
| We note that part of your methods state that patients were selected starting on 23 May 2016, whereas earlier in the methods April - June 2016 is cited. Please clarify the date your study began | We apologize for this mistake. The actual dates of interview and HBsAg sampling were from 12th April to June 17th. The mistake in dates have now been corrected. | Page 5 line 116 | Page 5 line 101 |
| Please provide more details on the limitations of your study methods. | The limitations of the HBsAg underestimating the prevalence is not unique to this, but also applies to similar prevalence studies in Uganda. The HIV specific population and treatment on tenofovir potentially lowering prevalence have been discussed in a paragraph under discussion. | Page 17 Lines 295-300 | Page 16, line 259-264 |
| We note that you state "CD4+ cell counts were significantly lower in the HBV patients (p=0.025)" however Table 2 shows the opposite. Please revise your table if, for example, values were swapped accidentally. | Thanks for noting this. However, the only time that CD4 was higher for the HBV positive than the HBsAg negative was at baseline. In all the other times after ART initiation, the CD4_+ cell count was higher for the HBsAg positive. The p value of 0.025 was noting that the rise in CD4+ count from baseline to current level is higher for HBsAg negative participants. No change has been made. | Table 2 | Table 2 |
| Please include additional information regarding the survey or questionnaire used in the study and ensure that you have provided sufficient details that others could replicate the analyses. For instance, if you developed a questionnaire as part of this study and it is not under a copyright more restrictive than CC-BY, please include a copy, in both the original language and English, as Supporting Information. | The two versions of the questionnaire have been added as supporting information | S3_ questionnaire  S3b File_translated_ questionnaire | S3 supporting information questionnaire files |
| In your Data Availability statement, you have not specified where the minimal data set underlying the results described in your manuscript can be found. PLOS defines a study's minimal data set as the underlying data used to reach the conclusions drawn in the manuscript and any additional data required to replicate the reported study findings in their entirety. All PLOS journals require that the  minimal data set be made fully available. For more information about our data policy, please see  http://journals.plos.org/plosone/s/data-availability | We thank the Editor for these comments.  We had earlier submitted the data as additional supporting information, in line with Plos recommendations. In this resubmission, we attach the final combined dataset as supporting information. However, we have not deposited it in any other specific online location. | S2_dataset | S2_dataset |
| Upon re-submitting your revised manuscript, please upload your study’s minimal underlying data set as either Supporting Information files or to a stable, public repository and include the relevant URLs,  DOIs, or accession numbers within your revised cover letter. For a list of acceptable repositories,  please see http://journals.plos.org/plosone/s/data-availability#loc-recommended-repositories. Any potentially identifying patient information must be fully anonymized | Specific identifying materials have been removed and dataset attached. | S2_dataset | S2_dataset |
| The population size is good at >950 individuals. The overall prevalence of 7.9% is what would be expected for a country in sub-Saharan Africa. However, this manuscript would benefit from careful review by a native English speaker and/or a professional editing service. | We thank you for the kind comment about sample size. Our point in this paper is that for the specific higher burden Northern Ugandan region. The described prevalence is lower than previous findings.  Proof reading (not professional editing) has been done leading to changes in sentence constructions, flow and edits in the document. We have duly acknowledged this service | All sections of the document  Page 18, line 324 | All sections of the document  Page 18, line 286 |
| The authors should clarify what population reference 10 was conducted in . . . was this the general population or persons living with HIV? | The referenced study was done in the general population, with and without HIV. A statement “with no documentation of HIV status” has been added | Page 4 line 78 | Page 3 line 69 |
| Was the data collection questionnaire self-administered or conducted by a researcher or clinician? | The questionnaires were administered by nurses trained as research assistants. All of them had experience in HIV care. A statement to that effect has been added | Page 6 line 121 | Page 4 line 106 |
| How was HIV treatment adherence reported and confirmed? | Adherence was measured using a composite of self-reporting and pill count, or recorded as assessed and documented by the primary health provider |  |  |
| The authors should comment on how many individuals receiving ART were receiving HBV-active drugs as part of their ART regimen. These data on specific ART regimens is confusing. Tenofovir is mentioned but what about 3TC? Are they always given together or could some individuals receive tenofovir only or 3TC only? | The Primary backbone regimen in use in Uganda has been Tenofovir/Lamivudine fixed dose combination, usually in addition to EFV or NVP. All the patients an ART the clinic actually take Lamivudine in combination with other drugs. We did not consider Lamivudine combined with Abacavir or Zidovudine to be an optimal treatment- as guided by the Uganda HIV treatment guidelines. 86.84% percent were on Lamivudine/Tenofovir Diopropyl Fumerate and 13.16% were on Lamivudine combined with zidovudine or Abacavir, as part of their Antiretroviral treatment.  Finally, we did not mention treatment with lamivudine-only because of the low rate of HBsAg seroconversion seen with this regimen, which may not change the results of our study | Note ++ to table 2 | Note ++ to Table 2 |
| It appears that HBV DNA testing was not conducted. This and the lack of information on HBV genotypes should be mentioned explicitly as limitations in the discussion. | Given the prohibitive cost of HBV DNA testing and the high number of positive respondents, we were unable to provide testing. We have added a statement about the potential better performance of HBsAg in high HBV viraemia, as a limitation under Discussion section | Page 17 Lines 295-300 | Page 16, line 259-264 |
| REVIEWER 1 |  |  |  |
| The study speaks of a decline in HBV prevalence based on comparison with a previous population based study. However, there were differences in the studied populations. The population in the recent study was mostly on Tenofovir a potent anti-HBV drug for a median duration of 6 years. It is expected hat the prevalence will be lower due to treatments effects as some patients might have lost the  HBsAg. The population based 2010 survey might have included HIV negative participants who were not on HBV active treatment or people living with HIV but not on HAART. The ‘decline would have been better ascertained if it was compared to previous treatment experienced patients in the same population. Furthermore, the study admits to the possibility of the HBsAg underestimation due to the  kit used, which was not reviewed for performances in low HBV levels. The prevalence reported was also similar to studies in similar populations elsewhere | The assertions about a decline in prevalence compares many previous studies to current ones, not just the 2010 study, as articulated under discussion, lines 225 to 229.  As reported in the “Discussion” paragraph, we are aware that it is not possible to compare a prevalence study conducted on general population with one conducted on an HIV-positive group. However, data about Northern Uganda are still few, so we had to use all the available sources.  We discussed why our population may have biases with the general population that explains this result (gender disproportion, we excluded all patients with critical conditions, we recruited potentially more adherent).  Although it is true that long term TDF therapy may lead to HBsAg seroconversion, as stated in 2017 EASL guidelines this is a really rare event (3% after 48-52 weeks of TDF treatment in HBeAg positive patients and 0% after 48-52 weeks of TDF treatment in HBeAg negative patients). The bias is possible but of minor entity. Thus the decline may not be negligible | Page 15, lines 240-252 | Page 14 lines 211-221 |
| MINOR REVISIONS  Abstract:  2. People living with HIV/AIDS (PLWHA) should be changed to ‘People living with HIV (PLWH)’  3. Study type and time of enrolment is missing  4. HbsAg under Patients and Method should be changed to HBsAg  5. Shored in results section should be corrected to shared  6. CD4+ cell should be corrected to CD4+ T cell here and elsewhere in the article. | 2. Thanks for the suggestion. This has been corrected in 10 instances and now appears as PLWH  3. Study type: cross sectional study, and period (April to June) now included. This has been corrected, page 2 lines 30 and 33  4. Thanks for this. Four instances were corrected in text, and multiple instances in the tables.  5. Shored now appears as shared  6. Thanks for the suggestion. We have corrected multiple incidences in the text and tables | Lines 25, 30, 46, 62, 98, 154, 278, 281, 283  Page 2 lines 30 and 33  Page 2 line 32 and others  Page 2 line 39  Page 2 lines 40 and 42, and others | Page 2 lines 30 and 33  Page 2 line 31  And others  Page 2 line 38  Page 2 lines 39 and 41 and others |
| **Introduction**  7. WHO should be written in full since it’s first mention.  8. There is a space missing between the sentence (page 3) Uganda was thetheatre of a civil war from 1989 to 2006’ and the reference.  9. HIV clients should be changed to people living with HIV (PLWH)  **Materials and methods**  10. Page 5, In the sentence A questionnaire was administered to collected information’, the word collected should be corrected to collect  11. Page 5, AST and ALT should be written in full at first mention.  12. Page 6: The authors mention that testing was done ‘in accordance with the standard procedures of St. Mary’s Hospital Lacor’. Are the procedures different from the manufacture’s protocols? If they are then the differences should be noted for ease of reproducibility since the hospital’s procedures were not referenced.  13. In the sentence ‘We also made CD4+ T cell counts’, made should be changed to measured | 7. Thanks, this has been corrected.  8. This space has now been included  9. Thanks for these, which have been corrected as shown under 2 above  10. We appreciate this comment. The word “collected” now appears a s “collect”  11. Corrected as suggested  12. The Standard operating procedures do not vary. We have now added “in accordance to the standard operating procedures of the manufacturers adapted at St. Mary’s hospital Lacor.  13. The word measured is now in place of made | Page 3 line 57  Page 4 line 74  Lines 25, 30, 46, 62, 98, 154, 278, 281, 283  Page 6, line 121  Page 6 line 133-134  Page 6 line 136  Page 6 line 137 | Page 3 line 52  Page 3 line 66  Page 5 line 106  Page 5 line 117-118  Page 6 line 120  Page 6 line 131 |
| **Results**  14. The authors mention that the 35 subjects excluded were not going to affect results but did not qualify the statement as to why/how they were not going to affect results  15. Include explanations of all abbreviations below tables.  16. Page 10. The authors states that ‘All of the variables with a P-value of >0.2 at univariate analysis were included in a multivariate analysis, but table 3 includes variables with p values which were at univariate analysis < 0.2  17. Table 4 is not uniform. Some variable are written in all capital letters while others are written insentence case. | 14. Because of the sample size, the results of a statistical analysis conducted on 985 or 950 subjects did not gave significantly different results. Consequently, we decide to present in the paper the analysis on the 950 patients undergoing ART to have a more homogeneous sample to discuss.  15. These have now been included where elsewhere missing.  16. Thanks for noting this mistake, which has now been corrected to p values <0.2%  17. Table 4 now corrected to have uniform non capitalized letters | Tables 1,2,3  Page 11 line 224  Table 4 | Tables 1,2,3  Page 10 line 181  Table 4 |
| **Discussion**  The discussion is well written with sound conclusions. | Thanks for the encouraging comment |  |  |
| REVIEWER 2 |  |  |  |
| MAJOR REVISIONS  In the Discussion section on page 14, the authors state the following;  “This leaves a new question to confirm: is there a true decline in HBV prevalence in the region?”.  To better address the hypothesis of the study, could the authors clearly comment on how the following could have impacted on the prevalence of HBsAg found within the study population:  •The study made use of a health facility-based population that may have different health-seeking behaviour from PLWHA within the general population. In addition, the fact that 86.8% of the study population were on long-term HBV active regimens (3TC and TDF) could reduce the prevalence of HBsAg.  •It is well established that the burden of occult HBV infection (OBI) is higher among PLWHA than the general population. Given that the prevalence of OBI was not assessed as part of this study, could the  burden of HBV infection have been underestimated – could the lack of testing for anti-HBc and HBV DNA been a limitation to fully understanding the proportion of PLWHA who had not had been  infected with HBV? | Similar concerns have been answered under response to major revisions by Reviewer 1.  Most of the discussion focuses on the prevalence found.  The seeming decline in prevalence seen in this study is not an isolated finding. First, the population prevalence of HIV at the time was about 8.3%, and this is from the whole region.  Because of the low rate of HBsAg seroconversions anticipated with 3TC/TDF, our result should not be heavily biased by those therapy.  Of course the lack of data about HBV-DNA and HBcAb is a limitation, but  even when we anticipate OBI to reduce prevalence in HIV, the prevalence is actually lower than previous (Bwogi et al, Ochola et al), but higher than a similar study done around the same time in the same region (UPHIA report).A previous study conducted among women attending antenatal care (ANC) of the same area found HBV positive among 10.81% HIV positive pregnant women. Such leaves the question on decline as something to be confirmed.  Lack of HBV DNA testing has been noted as a limitation answered above under the last comment by the Editor, as well as to Reviewer 1. | Page 15, lines 240-252  Page 17, lines 292-300 | Page 14 lines 211-221  Page 16, lines 259-264 |
| MINOR REVISIONS  **Abstract**  1. “…selected to undergo a rapid hepatitis B surface antigen (HbsAg) after administering a questionnaire.” Insert the word “test” after “HBsAg” for better clarity.  2.“…and having shored housing with HBV-infected people…” Do the authors mean “shared”? | 1.Thanks for the suggestion, “Test” has been inserted  2.The right word “shared” now appears in place of shored | Page 2 line 32  Page 2 line 39 | Page 2, line 31  Page 2, line 38 |
| **Introduction**  3.Page 3; "About 5-15% of persons living with HIV worldwide have hepatitis B virus (HBV) infection".  The current Global Hepatitis Report (2017) compiled by the WHO estimates that the global prevalence of HBV infection in HIV-infected persons is 7.4%. I would suggest this as a more appropriate and upto-date reference.  4.Page 3; “…and a high prevalence in Chad, Cameron, and western Africa (≥8%)…” Do the authors mean “Cameroon”?  5. Page 4; “…co-infected subjects more rapidly develop liver fibrosis…and respond less to HBV vaccine.” Could the authors elaborate on this for better clarity? Given that the hepatitis B vaccine is a preventative and not a therapeutic vaccine, it would not be administered to those who are already infected. | We thank the reviewer for this suggestion, which has now been integrated in our references.  4. Thanks. We meant Cameroon, now correctly written  5. What we describe is how PLWH respond to HBV infection or HBV vaccine. We agree that there would be no point in giving HBV vaccine to people already infected by HBV virus. In order to eliminate this misunderstanding, we modified the phrase to exclude vaccination | Page 3 line 48,  Reference 1 line 435  Page 3, line 59  Page 4, line 87 | Page 3 line 48, Reference1, page 19, line 296  Page 3 line 55  Page 4 line 76 |
| **Results**  6.Page 9; “…almost all the recruited participants were receiving ART (950/985)…” In the methods sections, the authors clearly indicate that all 35 participants who were yet to initiate ART had been excluded from analysis.  7.In Table 2, does the line item “Months of therapy” refer to the number of months participants have been on ART or some other form of therapy?  8..Page 10; “…and this was independently associated a higher CD4+ T cell count at the time…” insert the word “with” after “associated”.  **Discussion**  9.Page 15; “…crowding and promiscuity than that associated with village life…” I would suggest that the authors replace the word "promiscuity" with "high risk sexual behaviour" if this is indeed what they are referring to.  10. Page 16; “Finally, although they were not involved in HBV transmission…customary for Acholi men to have more than one official wife, the latter because P. falciparum malaria is widespread in the area.” If these findings have no bearing on the burden of HBsAg or the risk of HBV transmission within the population, I would suggest that the authors provide some clarity as to why it has been highlighted in the discussion section. | 6. The opening statement of results section does clarify on the exclusion of the 35 from analysis. They are not included in Table 2  7. This has been corrected to read “months of antiretroviral therapy”, since it applies to duration on antiretroviral therapy  8. Thanks. “With” has now been included  9. Thanks for suggesting a more respectful word that has now replaced promiscuity  10. Malaria is thought to predispose people to having severe anemia that might necessitate blood transfusion, a risk factor. These unstudied potential risk factors do exist in the community, hence the mention.  We have added that severe anemia is a potential consequence of P. falciparum infection. This is to support the reason for transfusion-based HBV risk. | Tables 2, 3, 4  Page 12, line 215  Page 16, line 262  Page 18 line 310 | Tables 2, 3, 4  Page 10, line 188  Page 15, line 228  Page 17, line 273 |
