# Supplementary material for: Hepatitis B and HIV coinfection in Northern Uganda: Is a decline in HBV prevalence on the horizon?
Source: PLoS One. 2020 Nov 18;15(11):e0242278. doi: 10.1371/journal.pone.0242278 (PMC7673526; doi:10.1371/journal.pone.0242278)
Supplement: Attachment — Submitted filename: Response to reviewer comments.docx [file pone.0242278.s005.docx]

St. Mary’s Hospital Lacor,

P. o. Box 180, Gulu

27^th^ October 2020

The Academic Editor,

Plos One

Dear Dr. Jason Blackard,

RE: RESPONSE TO REVIEW COMMENTS ON HEPATITIS B AND HIV COINFECTION STUDY IN UGANDA (**PONE-** **D-20-20265R1)**

The team of authors is grateful for the critical eye that the Editor and reviewers had in reviewing our manuscript, and for the suggested minor changes. The reviewer’s welcome suggestions have been addressed, as shown in Tabular format here below. For the Editor’s concerns about the laboratory protocols, we have attached the basic study standard operating procedures, all in the context of routine hospital care. We did not however deposit such online.

| **ISSUE** | RESPONSE | **Tracked document** | **Final document** |
| --- | --- | --- | --- |
| Reviewer 2 |  |  |  |
| Line 32; “Factors associated with HBsAg positivity were analysed univariate...” Please insert the word, “using” before “univariate”. | We thank the reviewer for spotting this. We have now added the word “using” to make the statement understandable | Page 6  Line 132 | Page 6  Line 132 |
| Line 165; “viral load, below 20 copies/μ.…” Please confirm the unit of measure for the viral load assessment. | The unit of viral load is copies per milliliters (ml). Therefore, ml has now replaced µ. Thank you very much. | Page 9  Line 165 | Page 9  Line 164 |
| Lines 206-207; “…HIV infected patients in area busy hospital clinic in Northern Uganda...” This sentence is unclear to me, kindly rephrase for better clarity | Thank you for noting this. The word area was supposed to be only “a”. The phrase now reads, “This study found that HBV infection prevalence among HIV infected patients in a busy hospital clinic in Northern Uganda was 7.9%” | Page 14  Line 206 | Page 14  Line 205 |
| Editor |  |  |  |
| Deposit laboratory protocols if applicable | This might not be applicable, We have however attached here below the study standard operating procedures and brochures for the tests by the manufacturers. |  |  |
| Not listed |  |  |  |
| There is a redundant full stop(.) after negatively on line 161 | Doubled full stop has been deleted | Page 9 line 161 | Page 9 line 161 |

Thank you again as we await any further needed clarification

Sincerely yours,

Dr. Emmanuel Ochola (On behalf of the Authors)

**STANDARD OPERATING PROCEDURES**

*Phlebotomy procedures*: trained personnel will execute the whole procedure. Phlebotomy will be performed following the standard procedure of the hospital.

*GPT (ALAT) liquiUV – System Reagent for HumaStar 600* and *GOT (ASAT) liquiUV – System Reagent for HumaStar 600* produced by Human Gesellschaft für Biochemica und Diagnostica mbH (Wiesbaden):

- collect whole blood in an EDTA tube
- pipette in cuvette at 37°C 100 µl of whole blood and 1000 µl of working reagent
- use a Humalyzer2000/Humastar300/Humastar 600 machine to analyse the sample

*One step HBsAg Test*- produced by Intec Products, Inc. (Xiang):

- collect whole blood specimens following regular clinical laboratory procedure, heparinized capillary tubes must be used. Whole blood specimens should be used immediately
- bring all reagents and specimens to room temperature
- remove the test strip from the foil pouch and place on a clean dry surface
- identify the test card for each specimen or control
- apply at least 80 ul of the specimen to the sample pad behind the mark at the bottom of test strip
- interpret the results at 15 minutes

The result has a sensitivity of 98,89%, a specificity of 98,87%, a predictive value of a positive result of 97,80%.

*CD4 count by BECKTON DICKINSON’S FACSCOUNT* : samples will be collected and analysed by the laboratory of the St. Mary Hospital following the standard procedure of Lacor facility.

*HIV-viral load*: samples will be collected on specific dried blood spot (DBS) cards and analysed by certificate personnel in a certified laboratory in the capital, Kampala, as per standard HIV care.

**HBsAg rapid testing brochure**

**AST/ALT testing brochure**
